# Rediscovery of *Nuvol umbrosus* Navás (Neuroptera, Chrysopidae, Leucochrysini): a redescription and discussion

**DOI:** 10.3897/zookeys.519.5996

**Published:** 2015-09-01

**Authors:** Catherine A. Tauber, Francisco Sosa

**Affiliations:** 1 Department of Entomology, Comstock Hall, Cornell University, Ithaca, NY 14853 and Department of Entomology & Nematology, University of California, Davis, CA 95616, U.S.A.; 2 Museo Entomológico “José Manuel Osorio”(MJMO), Universidad Centroccidental “Lisandro Alvarado”, Barquisimento, Lara, Venezuela.

**Keywords:** Chrysopinae, Leucochrysini, generic assignment, interspecific variation, intraspecific variation

## Abstract

The monotypic leucochrysine genus *Nuvol* was previously known from three specimens of *Nuvol umbrosus* Navás, collected in the Atlantic Forest region of Brazil. For many years these specimens have been missing, and the genus has remained without a modern description. Here, the species is redescribed based on two newly discovered specimens (females) from the Amazonian region. The female terminalia are relatively simple, except for the subgenitale, which is enlarged, folded into two sections, and heavily sclerotized. Unique aspects of the wing venation and the unusual pattern of banding on the wings support the retention of *Nuvol* as a valid genus within the Leucochrysini. There are differences between the Amazonian specimens studied here and the earlier descriptions based on specimens from the Atlantic Forest. These differences may indicate the presence of two distinct, geographically separated species within the genus. However, largely because we do not know the sexes of the earlier specimens, we are treating the differences discovered in the two female specimens as expressions of intraspecific variation.

## Introduction

Leucochrysini (Neuroptera: Chrysopidae) is a large tribe of green lacewings restricted to the New World. Currently, it includes seven genera in varying states of systematic resolution. One of these genera, *Nuvol*, has received no modern taxonomic treatment: accurate drawings of the only included species, *Nuvol umbrosus* Navás, are available solely for the wings; its abdominal and genitalic features have not been described (Brooks and Barnard 1990). As a result, the generic distinction of this genus has remained in need of reevaluation for some time.

At the time of its description, the single type specimen of *Nuvol umbrosus* remained in the Navás collection in Barcelona (Navás 1916); it and one other specimen that Navás (1929a, b) cited later from the Museum in Hamburg are lost (Monserrat 1985). And, unfortunately, the heretofore only known recent specimen of *Nuvol umbrosus* [reported from the Museum of Zoology, São Paulo, Brazil, MZUSP; see Brooks and Barnard 1990] now also appears to be missing (C. A. Tauber & M. J. Tauber, pers. obs.). However, during 2014 the authors independently discovered two additional specimens of *Nuvol* – one (a female) in the collection of the Instituto Nacional de Pesquisas da Amazônia (INPA), Manaus, Brazil, and another (a female) in the Utah State University Collection, Logan, Utah. Both specimens are similar, and at this time we are treating them as examples of *Nuvol* ’s type species, *Nuvol umbrosus* Navás, 1916 (however, see below).

To help determine whether the genus should be retained as a valid entity within the Leucochrysini, the newly discovered *Nuvol* specimens are described and their features compared, including the taxonomically important genitalia, with those of other leucochrysines. Unfortunately, the generic placement of *Nuvol* will remain unresolved largely because our specimens are both females, and the male genitalia, as well as the larvae, are unknown.

## Results

### Tribe Leucochrysini

The tribe Leucochrysini contains the seven genera and two subgenera listed below. Following each genus name are: (i) the number of species currently included in the genus and (ii) references to recent systematic work dealing with the genus.

*Berchmansus* Navás, 1913 – (two species; Tauber 2007, Tauber and Tauber 2013)

*Cacarulla* Navás, 1910 – (monotypic; Brooks and Barnard 1990)

*Gonzaga* Navás, 1913 – (eight species; Brooks and Barnard 1990, Tauber et al. 2008)

*Leucochrysa* McLachlan, 1868

Subgenus *Leucochrysa* McLachlan, 1868 (41 species; Brooks and Barnard 1990, Tauber et al. 2013a, b)

Subgenus *Nodita* Navás, 1916 – (150 species; Brooks and Barnard 1990, Tauber et al. 2011a)

*Neula* Navás, 1917 – (monotypic, no specimens known; Brooks and Barnard 1990)

*Nuvol* Navás, 1916 – (monotypic; Brooks and Barnard 1990)

*Santocellus* Tauber & Albuquerque, 2008 – (two species; Tauber 2012)

The genus *Vieira* Navás, 1913, which was previously placed in the Leucochrysini (see Brooks and Barnard 1990), is now in the tribe Belonopterygini (Tauber 2007).

#### Genus Nuvol

Taxon classificationAnimaliaNeuropteraChrysopidae

Navás, 1916

##### Monotypic genus.

**Type-species**: *Nuvol umbrosus* Navás, 1916.

##### Known geographic distribution.

Brazil: States of Rio de Janeiro (RJ), São Paulo (SP), Amazonas (AM), Rondônia (RO), as follows. **RJ**: type specimen reported in original description, Rio de Janeiro, II-1912 (Navás collection, specimen missing); subsequent specimen reported from Prov. Rio de Jan., 20.X.1906, Coll. V. Bönninghausen. M. H., (Navás 1929a, b; Hamburg Museum, probably destroyed during WWII); **SP**: Alto da Serra, ii-II (or XII)-28, R. Spitz leg (examined by Adams’ at the MZUSP, without abdomen, specimen apparently missing); **AM**: Novo Aripuanã 05°15'53"S / 60°07'08"W. Armadilha Malaise em igarapé; Floresta úmida, ix.2004, Henriques Silva & Pena Leg; **RO**: 62 km SE Ariquemes, 7–18 Nov 1995, W. J. Hanson (USU)].

#### Nuvol umbrosus

Taxon classificationAnimaliaNeuropteraChrysopidae

Navás [1916.??.??]

Nuvol umbrosus Brotéria (Zoológica) 14: 14–35 (description). Navás 1929a: 860 (as “Newol” umbrosus, locality record); Navás 1929b: 319 (locality record); Penny 1977: 28 (species list); Brooks and Barnard 1990: 251 (taxonomy); Oswald 2013 (catalog listing).

##### Type.

The original description states that the type specimen was collected in “Rio de Janeiro, Febrero de 1912” and that it was retained in the Navás collection. The specimen is not reported to be in the Navás collection now (Monserrat 1995).

At some time, a neotype should be designated for this species. However, we are not doing so with either of the two extant specimens because: (1) They are both from the Amazonian region of Brazil, far removed from the type locality – the Atlantic Forest region of Rio de Janeiro. (2) Our specimens are both females; the sexes of the previously described species are unknown. (3) Our specimens both differ significantly from Navás’ illustration and description in aspects of the fore and hind wing venation (discussed below). Thus, until male and female specimens from near the type locality are available for comparison, we consider it prudent to withhold from designating a neotype.

##### Description.

**Figure 1.**
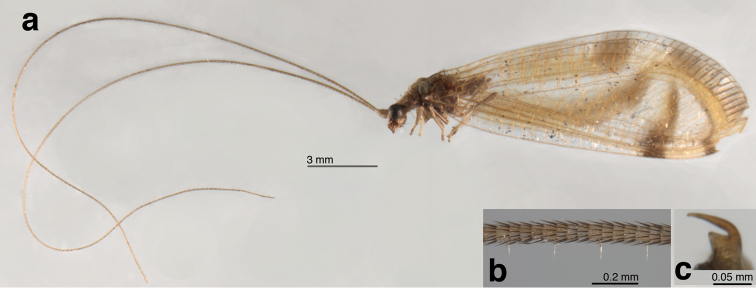
*Nuvol umbrosus* Navás adult: **a** habitus, lateral **b** antennomeres ~ 1/3 distance from base of antenna **c** mesotarsal claw (Brazil, Rondônia, CAT).

**Figure 2.**
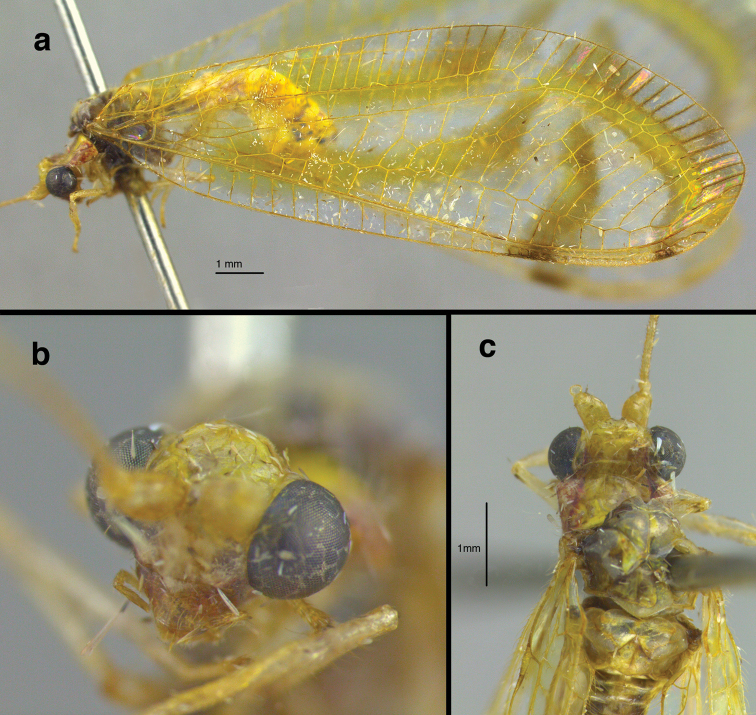
*Nuvol umbrosus* Navás adult: **a** habitus, lateral **b** head, frontolateral **c** head, thorax, dorsal (Brazil, Amazonas, FS).

**Figure 3.**
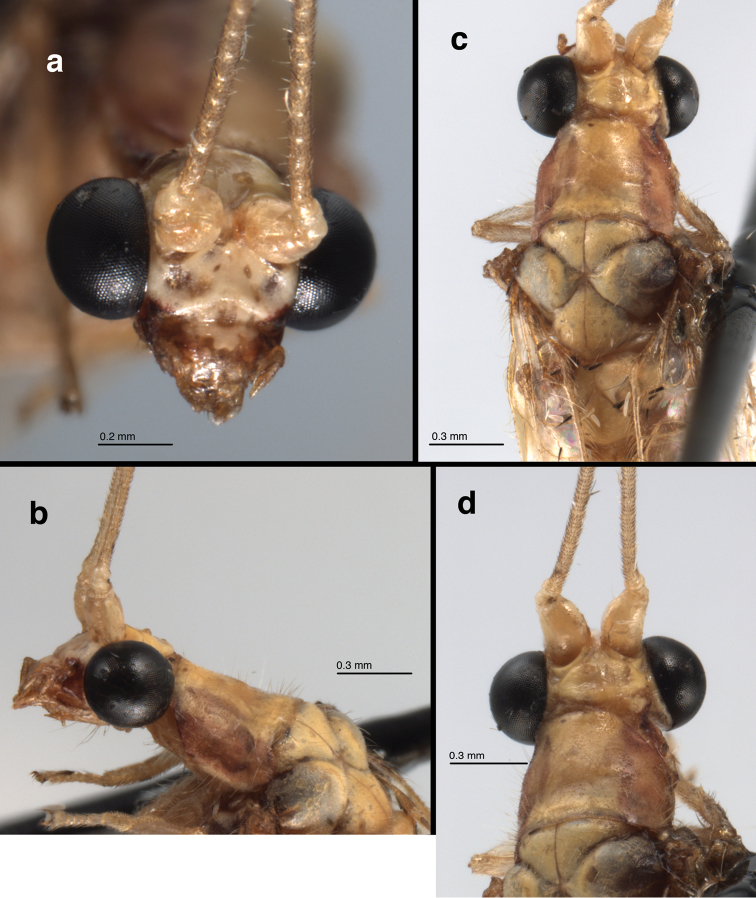
*Nuvol umbrosus* Navás adult: **a** head, frontal **b** head, prothorax, lateral **c** head, thorax, dorsal **d** head, prothorax, dorsal (Brazil, Rondônia, color faded, CAT).

**General** (Fig. 1): Body slender, yellowish or greenish brown, with elongate, slender antennae typical of leucochrysines, wings hyaline, marked with conspicuous brown to golden bands. **Head** (Figs 1, 2a, b, 3): Vertex yellowish green, with light reddish markings laterally, along eyes; surface slightly rough, somewhat raised posteriorly. Frons, clypeus white with lateral margins dark red; gena dark red. Labial palpus, maxillary palpus cream colored, without marks. Antenna cream colored, without markings. Scape elongate, relatively large, close mesally; flagellar segments (beyond basal section of flagellum) elongate, each with four swirls of robust, acute setae (Fig. 1b). Measurements: head width (dorsal) 1.7 mm, ratio of head width to eye width 2.0; distance between eyes (frontal) 0.78 mm; distance between tentorial pits 0.57 mm; length midantenna to midway between tentorial pits 0.53 mm; antenna length 31-35 mm; scape length 0.48 mm, width, 0.38 mm; basal two flagellomeres 0.1 mm long, 0.1 mm wide; flagellomeres at 1/3 distance to antennal tip 0.21 mm long, 0.07 mm wide. **Thorax** (Fig. 3c, d): Prothorax slightly wider than long (length 0.92 mm, width 1.27 mm), yellowish green mesally, with broad, red to brown longitudinal bands laterally, with elongate, golden setae laterally. Mesothorax, metathorax mostly yellowish green mesally, brown laterally, with fine golden setae dorsolaterally. Legs pale, without markings, with numerous light brown to amber setae; tarsal claws with broad, dilated base, deep narrow cleft (Fig. 1c).

**Figure 4.**
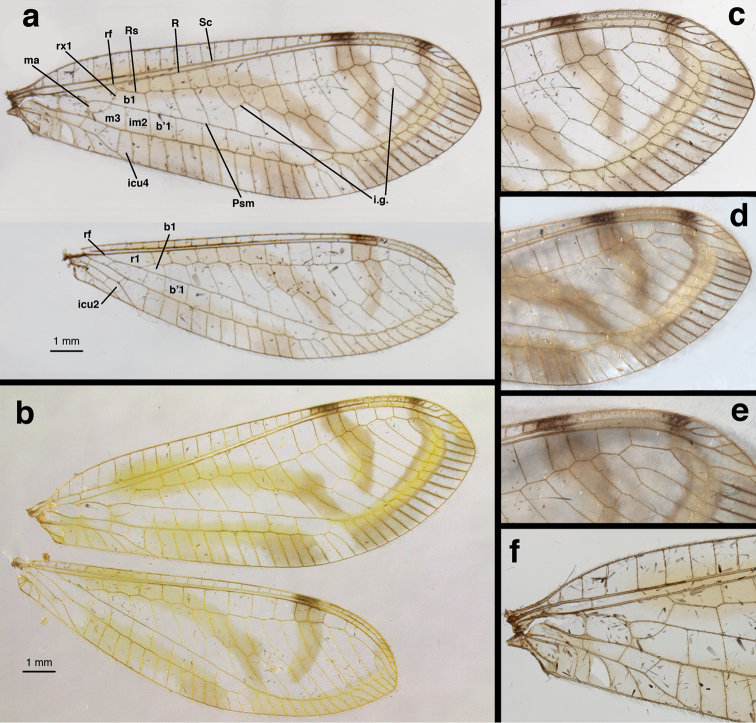
*Nuvol umbrosus* Navás wings: **a, c, d, e, f** Brazil, Rondônia, CAT **b** Brazil, Amazonas, FS. Note the unforked apical veinlets, markings, radius turning downward at tip, forewing with four intracubital cells. **b1** first upper Banksian cell **b’1** first lower Banksian cell **icu2, icu4** second and fourth intracubital cells **im2** second intramedian cell **i.g.** inner gradate series **m3** third median cell **ma** median arculus (base of first intramedian cell) **Psm** pseudomedia **R** radius **Rs** radial sector **rf** origin of radial sector **rx1** first radial crossvein **Sc** subcosta.

**Wings** (Fig. 4): Forewing 14.8–15.8 mm long, 5.1–5.5 mm wide (at widest point); ratio of length: maximum width = 2.9:1. Costal area relatively narrow; tallest costal cell (#6–8) 0.8 mm tall, 1.4–1.8 times width, height 0.17–0.15 times width of wing (midwing). First intramedian cell ovate, height (along median arculus) 0.33 mm, width 2.5–2.6 times height, 0.65–0.66 times width of third median cell. First radial crossvein distal to origin of radial sector (Rs); radial area (between radius and Rs) with single row of 15 closed cells; tallest cell (#6–8) 1.3–1.5 times taller than wide. Base of subcosta, upper media, lower media slightly crassate, other longitudinal veins robust; 4 b cells (= cells beneath Rs, not including an inner gradate vein). Seven to nine discrete inner gradates in irregular pattern, outer gradates apparently aligned with distal veinlets in smooth, curved line paralleling margin of wing from tip of pseudomedia (Psm) to tip of Rs. Height of fourth gradate cell 2.9–3.9 times width. Six to seven b’ cells (cells beneath Psm after second intramedian cell). Four intracubital cells (three or four closed). Subcosta, radial sector forked apically; almost all other apical veins unforked. Membrane with four large, conspicuous yellow to brown marks; stigma with dark brown marks basally, distally. Veins mostly pale, except under markings. Hindwing 13.1–13.8 mm long, 4.1–4.3 mm wide. Six discrete inner gradates, outer gradates similar to those of forewing; 13–15 radial cells (counted from origin of radius, not false origin). Five large b cells (no small “t” cell); six b’ cells beyond second intramedian cell; two intracubital cells (one closed). Membrane with yellow to light brown marks, similar to those on forewing; stigma with single dark brown mark basally. Veins pale except near stigmal marking.

##### Male.

Unknown.

##### Female

**Figure 5.**
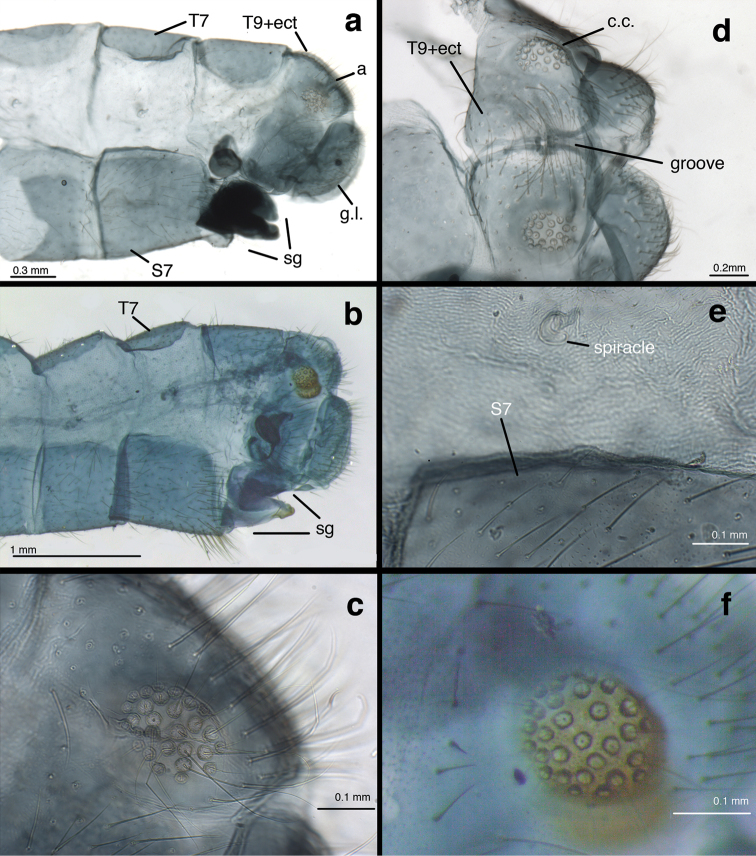
*Nuvol umbrosus* Navás female abdomen. **a, b** terminal segments, lateral **c** callus cerci and trichobothria **d** ninth tergite and ectoproct, dorsal, divided by deep groove **e** seventh pleuron and sternite, showing spiracle and texture of integument **f** callus cerci [**a, c, d, e**: Brazil, Rondônia, CAT; **b, f**: Brazil, Amazonas, FS]. **a** anus **c.c.** callus cerci **g.l.** gonapophysis lateralis **sg** subgenitale **sp** spermatheca **sp.d.** spermathecal duct **S7** seventh sternite **T7** seventh tergite **T9+e** fused ninth tergite and ectoproct.

**Figure 6.**
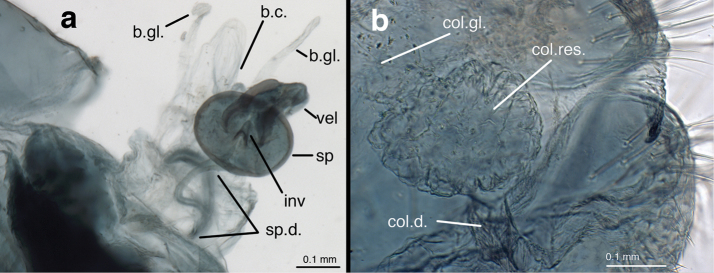
*Nuvol umbrosus* Navás female genitalia. **a** spermathecal complex **b** colleterial complex [Brazil, Rondônia, CAT]. **b.c.** bursa copulatrix **b.gl.** bursal gland **col.d.** colleterial duct **col.gl.** colleterial gland **col.res.** colleterial reservoir **inv** invagination **sp** spermatheca **sp.d.** spermathecal duct **vel** velum.

**Figure 7.**
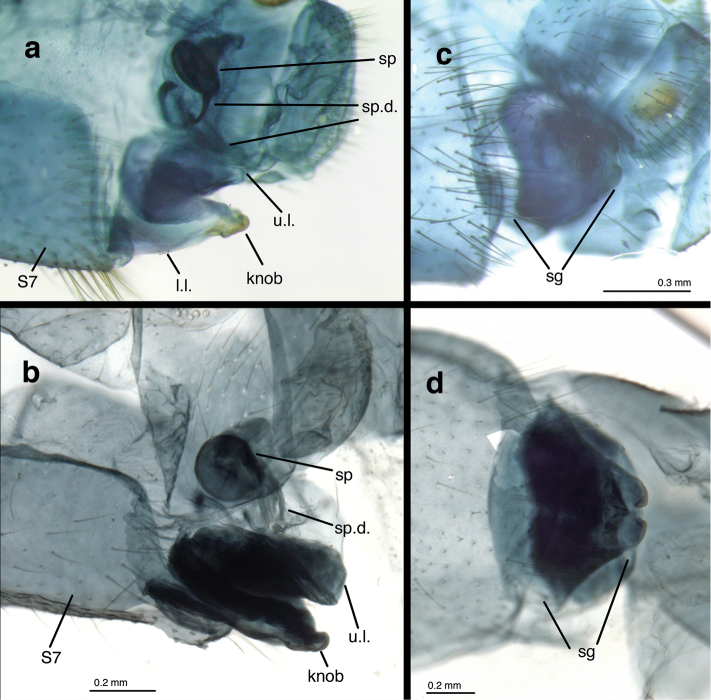
*Nuvol umbrosus* Navás female genitalia. **a, b** spermathecal complex and subgenitale, lateral **c, d** subgenitale, ventral [**a, c** Brazil, Amazonas, FS **b, d** Brazil, Rondônia, CAT]. **l.l.** lower layer of subgenitale **sg** subgenitale **sp** spermatheca **sp.d.** spermathecal duct **S7** seventh sternite **u.l.** upper layer of subgenitale.

(Figs 5–7): Abdomen with spiracles simple (Fig. 5e); callus cerci round, brown to black, heavily sclerotized (INPA specimen), located dorsally on T9+ect, with 28–32 trichobothria stemming from irregularly spaced sockets (Fig. 5c, f); ninth tergite (T9) and ectoproct fused, but invaginated distally (Fig. 5a); T9+ect completely divided dorsally by deep groove (Fig. 5d). Praegenitale absent. Colleterial complex (Fig. 6b) with elongate, delicate gland, apparently with scattered particles; reservoir sperical, with colliculate, membranous surface, extending apically only into ectoproct; transverse sclerite delicate, membranous, with small, elongate teeth. Spermathecal complex (Fig. 6a) simple; spermatheca pillbox-shaped with small to moderate, V-shaped invagination; spermathecal duct elongate (~2.5–3× width of spermatheca), curvy, covered with fine, hair-like, glandular ducts throughout distal ~2/3 of duct, longest and most dense distally, becoming thinner to absent basally; velum small, connecting directly to bursal duct; bursal duct leathery, elongate, bent, extending posteriorly to bursa copulatrix; bursa membranous, with two narrow glands. Subgenitale (Fig. 7) large, leathery, almost as broad as tip of S7, composed of upper and lower sections extending distally from relatively deep fold at tip of S7, well beyond sternite; lower section triangular (ventral view), slightly concave, with paired distal lobes extending posteriorly as a knob (lateral view), lobes separated by small, mesal groove; upper section of subgenitale dense, especially laterally, slightly convex, folded above and connected basally to lower section.

##### Larvae.

Unknown.

##### Biology.

Unknown.

##### Variation.

Other than differences in color, the two Amazonian specimens examined are very similar to each other, and we are confident that they are conspecific. The coloration of the specimen from Amazonas (INPA) appears deeper than the one from Rondônia (USU) that might be slightly teneral.

## Discussion

***Nuvol*’s generic relationships.** In the original description (Navás 1916) and in subsequent discussion (Brooks and Barnard 1990), *Nuvol umbrosus* was said to resemble *Leucochrysa*, but also to differ from it in several features of the wings: notably an elongate radial vein that parallels the costa and curves posteriorly at the tip of the wing, outer gradates aligned with the bases of the distal veins in a smooth line that parallels the wing edge, an elongate stigma, four intracubital cells in the forewing, and distinctive markings on the fore and hind wings. These characters were all present in the specimens described herein, and, other than the four intracubital cells that also are present in *Berchmansus* (Tauber 2007), they readily distinguish *Nuvol* specimens from those in other leucochrysine genera.

At this time when the anatomy of females from only a small percentage of leucochrysine species has been described, our study does not provide any novel insights into the relationships of this species with other leucochrysines. The internal genitalic structures of the *Nuvol* female are relatively simple, and they lack the complex, coiled bursal duct of *Berchmansus*. Thus, they resemble those known for females in a large number of other leucochrysine species. However, the heavy sclerotization and folding of the subgenitale are conspicuous and may be distinctive among leucochrysines. Thus, at this time, there is no compelling evidence that supports a change in the generic designation of *Nuvol*, and we consider that the external features above provide substantive evidence for a generic difference between *Nuvol* and other leucochrysines. We await the discovery of *Nuvol* larvae and broadly based comparative adult morphological and/or molecular studies of the Leucochrysini before making any generic level changes.

**Specific identity.** It is noteworthy that the two Amazonian specimens we studied differed somewhat from the images and descriptions of the Atlantic Forest specimens studied by Navás (1916) and Adams (via Brooks and Barnard 1990). Specifically, the degree of supression in the forking of the apical veinlets is much stronger in the Amazonian than in the Atlantic Forest specimens. Illustrations by Navás (1916, fig. 6) and Adams (fig. 519 in Brooks and Barnard 1990) of the Atlantic Forest species show forewings with 16 and 14 forked apical veinlets; only four or five of the anterior radial veinlets are unforked. However, in the Amazonian specimens none or only one of the apical veinlets is forked. The difference also occurs in the hind wings. In addition, there seem to be some differences in the head and thoracic markings between our specimens and those described by Navás.

Although it is quite possible that the above venational and color differences are expressions of interspecific variation, at this time we cannot exclude intraspecific, i.e., geographic or sex-associated variation. Our two Amazonian specimens are females; whereas the sexes of the specimens from the Atlantic Forest are unknown. And we note that leucochrysines are notorious for polymorphisms in body color and markings (Mantoanelli et al. 2006, Tauber et al. 2011b, Tauber et al. 2013a, b). Thus, until the discovery of additional specimens, the two studied herein should be considered as variants of one species.

### Modifications for Brooks and Barnard’s (1990) key to adults of chrysopid genera

In the most recent taxonomic key for chrysopid genera (Brooks and Barnard 1990), *Nuvol* females could be recovered with small alterations to couplets 82 and 83, and the addition of another couplet, as follows below. Please note: Except where noted, the figure numbers below are those of Brooks and Barnard 1990).

**Table:** 

82	Ectoprocts not fused dorsally, or with deep dorsal groove (Fig. 22; also see Fig. 5d here); forewing usually marked with black spots, particularly on cell *dcc* or with four large, brown bands; longitudinal veins often unforked apically	**82A**
–	Ectoprocts fused dorsally; forewing usually unmarked; longitudinal veins forked apically	**84**
82A	Ectoprocts separated by dorsal suture; forewing with black spots on *dcc* and pterostigma (Fig. 280); claws with basal dilation (Fig. 11); basal lobe of subgenitale with V-shaped indentation (Fig. 286)	***Chrysemosa* Brooks & Barnard**
–	Ectoprocts separated by deep dorsal groove; forewing unmarked or marked with numerous small black spots or four large brown bands throughout the wing (Figs 456, 519); claws with or without basal dilation; basal lobe of subgenitale without V-shaped indentation	**83**
83	Forewing unmarked or marked with numerous small black spots throughout wing (Fig. 456); claws undilated (Fig. 12); basal lobe of subgenitale elongate (Fig. 460)	***Suarius* Navás**
–	Forewing marked extensively with large brown bands (Fig. 519; also see Fig. 4 here); claws dilated (Fig. 11; also see Fig. 1c here); basal lobe of subgenitale broad, flat, heavily sclerotized (Fig. 7 here)	***Nuvol* Navás**

## Supplementary Material

XML Treatment for Nuvol

XML Treatment for Nuvol umbrosus
